# Investigating the Dynamic Aspects of Drug-Protein Recognition through a Combination of MD and NMR Analyses: Implications for the Development of Protein-Protein Interaction Inhibitors

**DOI:** 10.1371/journal.pone.0097153

**Published:** 2014-05-27

**Authors:** Massimiliano Meli, Katiuscia Pagano, Laura Ragona, Giorgio Colombo

**Affiliations:** 1 Istituto di Chimica del Riconoscimento Molecolare, CNR, Milano, Italy; 2 Istituto per lo Studio delle Macromolecole, CNR, Milano, Italy; Jacobs University Bremen, Germany

## Abstract

In this paper, we investigate the dynamic aspects of the molecular recognition between a small molecule ligand and a flat, exposed protein surface, representing a typical target in the development of protein-protein interaction inhibitors. Specifically, we analyze the complex between the protein Fibroblast Growth Factor 2 (FGF2) and a recently discovered small molecule inhibitor, labeled **sm27** for which the binding site and the residues mainly involved in small molecule recognition have been previously characterized. We have approached this problem using microsecond MD simulations and NMR-based characterizations of the dynamics of the apo and holo states of the system. Using direct combination and cross-validation of the results of the two techniques, we select the set of conformational states that best recapitulate the principal dynamic and structural properties of the complex. We then use this information to generate a multi-structure representation of the **sm27**-FGF2 interaction. We propose this kind of representation and approach as a useful tool in particular for the characterization of systems where the mutual dynamic influence between the interacting partners is expected to play an important role. The results presented can also be used to generate new rules for the rational expansion of the chemical diversity space of FGF2 inhibitors.

## Introduction

Protein-protein interactions (PPIs) are the key nodes of cellular circuitries underlying the regulation of most biological processes. Therefore, they represent an important class of targets for the development of novel human therapeutics. However, developing drug-like antagonists that engage protein-protein interaction sites has turned out to be highly challenging for a number of causes.

The solvent accessible area and shape of protein-protein interfaces represent the first hurdle. It has been estimated that on average an approximate SASA of 750 to 1500 A^2^ is buried on each side of the interface. Analysis of the structures of protein pairs have shown that the interacting surfaces are rather flat and lacking the typical small, deep cavities that are targeted by small molecules directed towards enzyme active sites [1], [2]. Moreover, in many protein-protein complexes, the complementary combination of the two interacting surfaces involves a high degree of flexibility and dynamics [3], [4]. In this case, there might be a subset of conformations on (one of) the targeted interfaces that can favorably recognize and bind a small molecule, which might not be immediately evident from the analysis of single crystal structures [4], [5]. The optimization of leads in this case requires a framework shift with respect to the classical approaches used for the improvement of the activities of, e.g., active-site targeting enzyme inhibitors. In the latter case, the small molecule is bound to the target in one preferential conformation, which is optimally represented by one single structure of the complex: possible pockets that can be reached and favorable interactions that can be established with the target are in general evident and are used to guide the addition/modification of functional groups on the starting scaffold in drug design efforts.

If the aim is the design of leads targeting large, flat, exposed and dynamic surfaces one should in principle consider different arrangements of the small molecule on the protein as well as different conformations of the protein binding site. To this end, approaches that permit the characterization of multiple, different dynamic conformational substates at atomic resolution may represent valuable tools in the development of new strategies for the design of molecules targeting protein-protein interactions [6].

In this context, theoretical methods based on equilibrium Molecular Dynamics (MD) simulations can be used to characterize both the range of alternative states that can be sampled by a ligand on the surface of a protein under specific conditions and the dynamics of the processes of conformational transition between different substates [7]–[9]. Experimental methods based on NMR spectroscopy can be used to investigate different aspects of protein dynamics in solution and their response to ligand binding. Local and long range perturbations induced by the ligand may have minor effects on the protein structure that can escape direct structural observation by NMR. NMR spectroscopy provides however, through relaxation measurements, a unique tool for a detailed characterization of changes in the protein internal motions and shifts of the populations of interconverting conformers, induced by ligand binding [10].

Combining the information obtained by the two approaches has the potential to provide relevant novel insight into the structural and conformational properties of dynamic complexes, such as those formed by large, flat and flexible protein surfaces and small molecules aimed at breaking protein-protein interactions. A notable example of this approach is the work of Dibenedetto et al. who used MD trajectories to help interpret two-dimensional (2D) NMR data, shedding light on the interaction between the extremely flexible target human α-synuclein and dopamine, to inhibit fibril formation [11].

**Figure 1:**
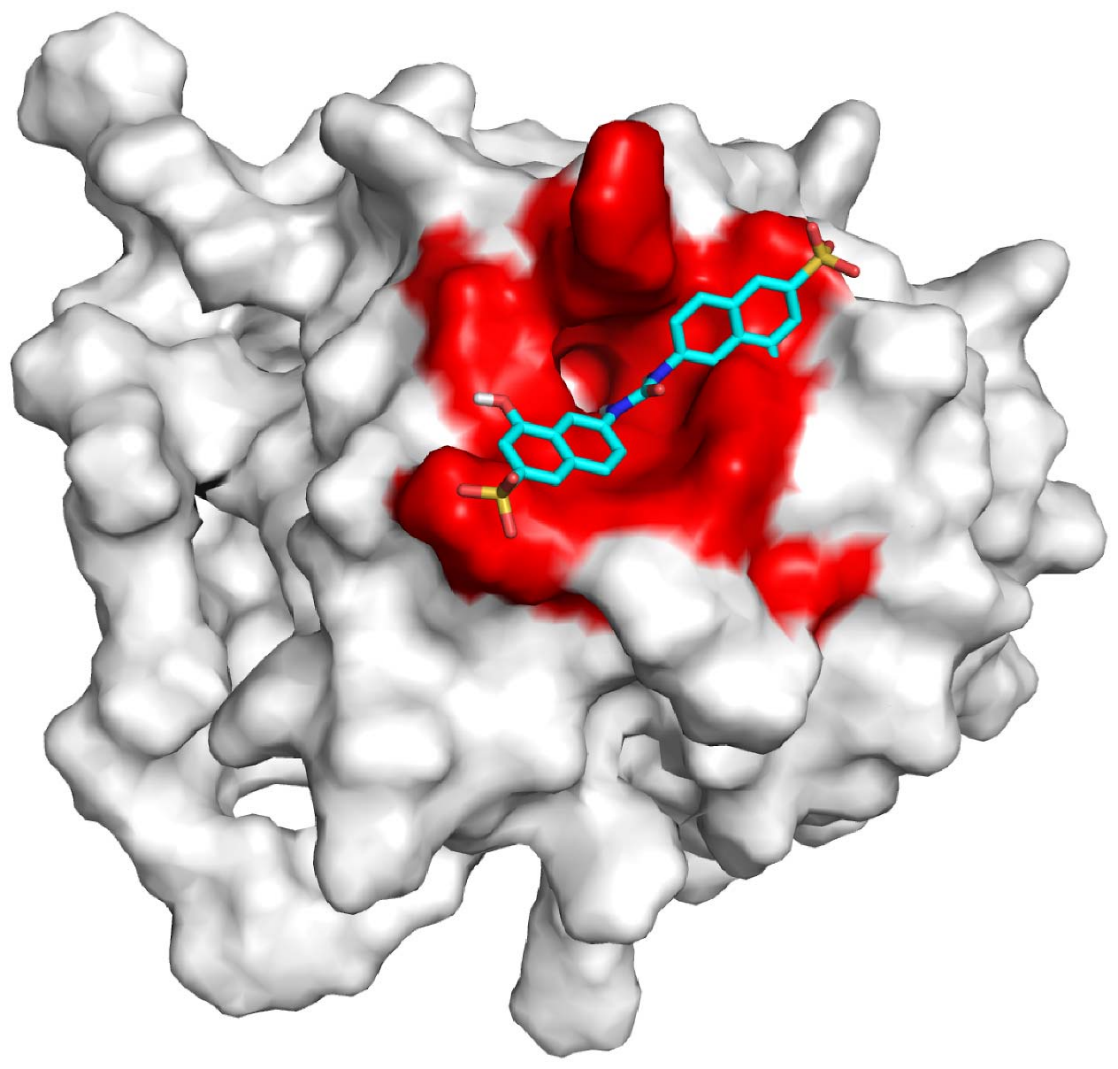
3D representation of the sm27/FGF2 complex. NMR-constraints derived structure of the complex between **sm27** and FGF2, used as a starting structure for all simulations and analyses. Red surface represents protein region involved in ligand recognition.

In this paper, we have investigated the complex between the protein Fibroblast Growth Factor 2 (FGF2) and a recently discovered small molecule inhibitor, labeled **sm27** (IUPAC name 4-hydroxy-6-((((8-hydroxy-6-sulfo-2-naphthyl)amino)carbonyl)amino)-2-naphthalenesulfonic acid) (Figure S1 in File S1)[12], [13]. The possible binding site on FGF2 and the residues mainly involved in small molecule recognition were previously investigated, suggesting that **sm27** engages the heparin-binding site of FGF2 (**Figure 1**). NMR and MD simulations revealed long-range allosteric effects, induced by the presence of the small molecule, on the FGF2 residues involved in the recognition of the cell receptor FGFR1 [14]. The direct and allosteric inhibition mechanism was demonstrated by SPR and cell-based binding assays, showing that upon **sm27** binding the formation of the ternary complex FGF2/HSPGs/FGFR1 is impaired.

While providing interesting mechanistic insight on the possible reasons at the basis of the small molecule antiangiogenic activities, analysis of the NMR data indicated that it was not possible to perform a classical NMR structural calculation for the **sm27**-FGF2 complex. Indeed the small number of FGF2 residues experiencing a significant chemical shift perturbation upon **sm27** binding, and the lack of an adequate number of protein-ligand NOEs precluded an accurate structure determination of the complex. These observations suggest the highly dynamic nature of the recognition, where the complex explores different substates.

To gain atomic resolution insights into the structural variation and conformational dynamics of the complex, we have combined the results of the analysis of microsecond-long MD simulations with NMR-derived measures of the structural, flexibility and hydration features of FGF2 in the *apo* and *holo* state (i.e. in complex with **sm27**). To this end, we have employed both established and recently developed methods of analysis of MD simulations. The aim is to define possible substates, in the microsecond trajectories, whose properties can account for the observed NMR-based experimental observables. This, in turn, is expected to provide a set of cross-validated and cross-filtered atomic resolution models of the main complementary interactions in the complex, which cannot be necessarily recapitulated by one single structure, and of their effects on the structural dynamics of the protein receptor. The emerging picture of the dynamic nature of the complex will be discussed in terms of its possible relevance to the design of novel protein-protein interaction modulators.

## Materials and Methods

### MD simulations and analyses

The apo structure of FGF-2 was obtained from the Protein Data Bank, code 1BLA.pdb. The starting structure for the **sm27**-FGF2 complex (holo system) was obtained as described previously, using the HADDOCK2.0 software and AIR deduced from chemical shift perturbation data [14].

The apo or holo systems were subsequently solvated in a cubic box large enough to contain 1 nm of water around the protein. The ε-amino groups were considered protonated and the carboxyl groups were considered to bear negative charges.

All MD simulations were performed using the AMBER 12.0 package [15] with the ff03 force field, the TIP3P water model [16], and the Particle Mesh Ewald summation method (PME) to deal with long-range Coulomb interactions [17]. The timestep for the integration of the equations of motion was 0.002 ps. Each system was simulated for 1 microsecond. A total of 100000 frames for analysis was saved for each system. All structural analyses were carried out using cpptraj/ptraj module as implemented in AmberTools 13 [15].

### Local flexibility

The analysis of local flexibility was performed based on the calculation of distance fluctuations as described in [8], [18], [19]. Briefly, we computed the matrix of distance fluctuations *A*:
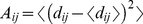
(1)where *dij* is the (time-dependent) distance of the Cα atoms of amino acids *i* and *j* and the brackets indicate the time-average over the trajectory. The *A* matrix, and various quantities derived from it, can be used to characterize the salient plasticity properties of a protein undergoing structural fluctuations in response to the presence (absence) of a ligand.

The local flexibility of a given amino acid, *i*, is defined with respect to its neighbors along the sequence, going from *i-2* to and *i+2* as 
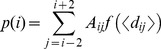
(2)where *j* runs over protein amino acids from *i-2* to and *i+2*, and *f* is a sigmoidal function that restricts the contribution to the sum to amino acids that are within about 7 Å from amino acid *i*: 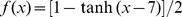. Highly flexible residues will be characterized by high values of *p*, because their local network of contacts appreciably changes due to the relative motion of neighboring residues, in the course of the dynamical evolution.

The average value of local flexibility per residue is obtained by averaging the values over the simulation time in each trajectory run:
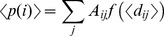
(3)


### S^2^ calculations

The analysis of the S^2^ parameter was carried out on the MD derived trajectories using the methods described by Bruschweiler *et al* [20] and implemented in analysis tools of the Amber suite.

### Protein hydration calculations

The hydration properties of protein residues on the surface was evaluated by calculating the mean residence time (MRT) of water molecules using the method proposed by Steinhauser *et al*. [21]. The detailed analysis of MRTs of water about proteins requires a rational criterion to discriminate whether a water molecule is a hydration or a bulk water. An easy and very fast way of classifying water molecules is based on distance. We consider a water molecule to belong to the first shell of hydration of the protein, and thus to be solvating a certain residue, when the distance between its oxygen atom to the closest nonhydrogen protein atom is less than 3.5 Å. This value corresponds to the first minimum of the radial distribution function of water around charged amino acids as described by Steinhauser *et al*.

MRT computation is based on the boolean variable *N_j_*(*t)*, which indicates whether a solvent molecule belongs to a specific shell *j* at time *t* or not, thus


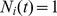
 if the molecule is in shell j;


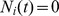
 otherwise

The MRT of a water molecule in shell *j* is obtained from the autocorrelation function *z_j_* calculated between *N_j_* at time *t* and at time *0*.

MRTs for water shells about individual residues. The MRT correlation function was calculated for all surface exposed residues. The MRT reference sites are the terminal nonhydrogen atoms in each amino acid. This analysis is implemented in the Amber tool available at the following website, http://www.stfc.ac.uk/CSE/randd/cbg/software/25249.aspx, which was used throughout the calculations.

### ^15^N relaxation experiments

R1, R2, and heteronuclear ^1^H→^15^N NOE, were measured for apo FGF2 at two magnetic fields (11.7 T, Bruker DMX 500 MHz and 14.1 T, Bruker DMX 600 MHz) at 298K to derive meaningful figures on backbone dynamics through Lipari-Szabo approach.

HSQC-based experiments were recorded to measure ^15^N longitudinal T1 and transverse T2 relaxation rates [22].

NMR spectra were acquired with a sweep width of 13 ppm and 40 ppm in proton and nitrogen dimensions, respectively. 1 k×128 data points were used.

At 500 MHz data-sets were collected with 64 and 96 transients per increment for T1 and T2, respectively. At 600 MHz data-sets were collected with 48 and 56 transients per increment for T1 and T2, respectively.

T1 values were measured using relaxation delays of 20 (x2 times), 60, 140, 240, 360, 520, 720, and 1500 ms for both 500 and 600 MHz. T2 experiments were recorded using delays of 15.5, 31 (x2 times), 46.6, 77.6, 124.2, 155.2, 186.2 and 217.3 ms for the 500 MHz, and 15.6, 31.2 (x2 times), 46.7, 77.9, 124.7, 155.8, 187, and 218.2 for the 600 MHz. The recycle delay between transients was set to 3 s for both T1 and T2 measurements.

For heteronuclear ^1^H→^15^N NOE experiments, interleaved proton-presaturated and non presaturated spectra were acquired. The interleaved spectra were separated by a Bruker standard macro. In the NOE experiment the recycle delay was extended to 4 s for both 500 and 600 MHz. The 2D spectra were recorded in phase-sensitive mode using time proportional increment [23] or the echo-antiecho method [24].

All spectra were processed with nmrPipe [25], intensities were calculated with nmrView (One Moon Scientific Inc). R1 and R2 relaxation rates were determined by fitting peak intensities to single-exponential, two parameters decay curves using the Rate Analysis tool inside nmrView. The Monte Carlo procedure was used to estimate the standard deviation of the data intensities. Relaxation data were obtained for 131 residues, out of the 146 possible correlations (FGF2 sequence contains 9 prolines) involving backbone amide protons. Resonance overlap was the major cause of difficulties in measuring peak intensities for the remaining 15 residues (M1, A2, F21, H25, L41, V49, Q65, G70, V71, S73, L91, V97, T121, W123, and T130). The results (Figure S2 in File S1) are in good agreement with data previously reported by us and others (see [14] and refs. therein).

For the analysis of the relaxation parameters the program Modelfree 4.20 by Palmer and co-workers [26] was used. Estimates of local correlation times for the NH vector of each residue were derived by analyzing R2/R1 ratios using r2r1_tm inside the program quadratic_diffusion (A.G. Palmer, Columbia University, New York). The overall correlation times were calculated as the average over the specific τ_m_ values by selecting only residues in β-strand elements, and excluding residues with steady-state ^1^H→^15^N NOE ≤0.6 and |(R2/R1) − 〈R2/R1〉| ≥SD [22]. Correlation times of 9.31±0.15 and 8.89±0.52 were estimated at 500 and 600 MHz, respectively. The relaxation data were analyzed with the axially symmetric model. Estimates for the rotational diffusion tensor and D_∥_/D_⊥_ were obtained using two programs: pdbinertia and quadratic_diffusion, both provided on A.G. Palmer's Web site (A.G. Palmer, Columbia University, New York). The ratios of three principal moments of the inertial tensor of FGF2 were estimated from its NMR solution structure (PDB-code 1BLA, [27]. Using the calculated rotational diffusion tensors, backbone relaxation data recorded at 500 and 600 MHz were simultaneously fit to the five standard Lipari–Szabo model-free formalism models [27]. Parameters of the model-free formalism were optimized for each residue individually, and the best parameter set identified by model selection according to d'Auvergne and Gooley [28] with Akaike Information Criterion (AIC). Of the 131 analyzed residues, 4 residues could not be fitted with any model: F26, R106, Y115, and I146.

A decrease of order parameters is observed in the region 3–29 and is indicative of high degree of flexibility. Excluding the mentioned residues, the average S2 value, calculated on 30–153 region is 0.84, consistent with a compact globular fold. Backbone slow motions, described through τ_e_ and Rex contributions, could also be derived for a subset of residues (Figure S2, S3 in File S1). The S2 values for the holo system have been previously reported [14].

### 1H-15N ePHOGSY experiments

2D ePHOGSY experiments with NOE/ROE step followed by ^1^H-^15^N HSQC detection [29] were acquired, combined with WATERGATE pulse sequence for water suppression. The pulse used for selective water excitation was a 50 ms long 180° Gaussian pulse. The mixing and spin-lock periods for NOE and ROE steps, respectively, were 80 ms long.

## Results and Discussion

A primary aim of this study was to examine whether it is possible to generate a simple molecular picture of the structural and dynamic effects determined by binding a drug-like ligand at a large, exposed surface of a protein, in the absence of a well defined binding site. In this case, one should consider that the single-structure representation commonly used in drug-design efforts based on X-ray crystal structures of protein-lead complexes may not be sufficient to recapitulate the main determinants of molecular recognition and binding. Therefore, a dynamic view of the complex could, on the one hand, provide a better understanding of the molecular reasons that determine the selection of a set of binding conformations among available states in the conformational ensemble, and on the other hand generate novel opportunities for lead improvement, revealing possible points of (dis)favorable interaction, hydration and flexibility modulation that can be exploited in the design of new drugs. This would complement existing efforts aimed at including protein flexibility in drug optimization.

There are thus several factors we focused our attention on, and that are described in the next sub-paragraphs.

### Comparison of S2 order parameters from NMR and MD

NMR relaxation parameters measured for the ^15^N-^1^H pair (longitudinal relaxation R_1_, transverse relaxation, R_2_, and ^1^H→^15^N NOE) can be combined within the Modelfree analysis, to obtain the amplitude of the order parameter, S^2^ [30]. *S* ^2^ reflects the amplitude of the fast internal motion of the H^N^-N bond vectors, in the picoseconds-nanoseconds timescale, and typically takes values between 0 (high motion, flexibility) and 1 (no motion, rigidity). Comparisons of MD simulations with NMR data on protein dynamics generally entails first the evaluation of the agreement between the experimental and MD derived *S* ^2^ [20]. In some cases, the comparison has been limited by the accessible timescales of the MD trajectories that turned out to be shorter than the overall tumbling correlation time. In this case, the access to microsecond-long simulations overcomes this limitation and allows a quantitative comparison between calculations and experiments.

**Figure 2:**
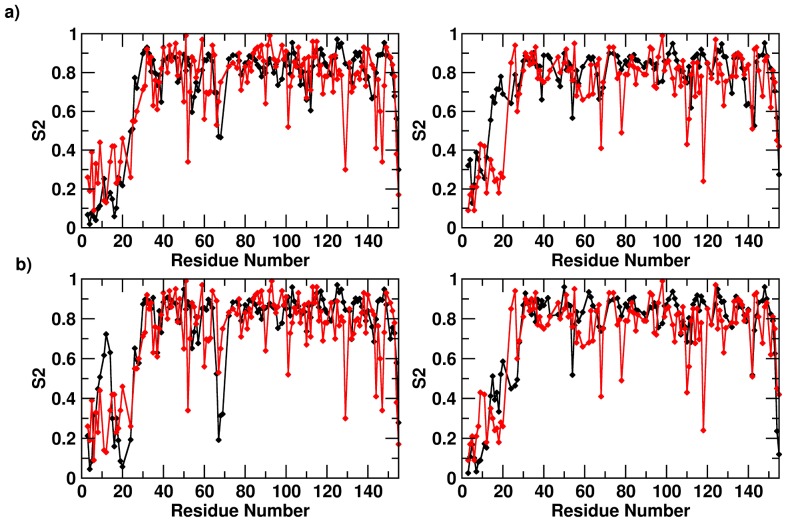
Comparison between the experimental and calculated *S^2^* values. The direct comparison between experimental (red) and calculated (black) *S^2^* parameters. Comparison between the two values for the apo (a) and holo (b) systems for *S^2^* calculated over the whole trajectory. Panel c) shows the comparison of experimental (red) *S^2^* parameters with values calculated over the 100 ns trajectory interval (black) that yield the best agreement (200–300 ns).

The analysis was thus carried out for the apo and holo forms of the protein. The results of the comparison are summarized in **Figure 2**. In general, the optimal agreement between the order parameters computed from the MD trajectory with the ones derived from NMR relaxation parameters (**Figure 2a, b**) suggests that the 1 microsecond-long MD simulations describe NH vector atomic motions with good accuracy in both the apo and holo systems. In order to define possible substates, in the trajectory of the **sm27**-FGF2 complex, whose properties can account for the observed NMR dynamic behavior, the microsecond long trajectory was subdivided into consecutive 100 ns intervals and the correlation between calculated and experimental S^2^ in each interval was re-estimated. The interval between 200 and 300 ns appears to yield the best correlation, with an R^2^ of 0.8 (**Figure 2**). This ensemble could define the suitable subset of interconverting conformational states for the generation of structural models that best fit the experimentally observed dynamic quantities.

### Insights on protein flexibility modulation upon ligand binding

The structural and dynamical consequences of complex formation at the residue level were further investigated with the aim of defining the specific regions of the protein that are most influenced by the presence of the ligand. The choice of a certain ensemble of conformations as the best representatives of the dynamic **sm27**-FGF2 complex was performed comparing the MD local flexibility parameter (Eq. 2 of Material and Methods) with ^1^H→^15^N NOE measurements, which gives information about the motions of individual NH/bond vectors in the ps to the ns timescale. The rationale of using NOE to refine the conformation selection process is that NOE is an observable quantity, that is a property that can be measured directly without any assumption and approximations, which are instead needed in the case of S^2^ order parameters [31].

Local flexibility, defined as average deformation that is locally experienced by each residue in a given (un)bound state, can be considered as a simple approximation to the value provided by ^1^H→^15^N NOE measurements, which are averages over the ensemble of the visited protein states. Residues undergoing motions faster than the protein molecular tumbling show a decreased ^1^H→^15^N NOE intensity relative to the observed average value. MD local flexibility data were obtained by calculating the time-averaged local flexibility over the whole MD run and on consecutive intervals of 100 ns each (0–100 ns, 100–200 ns…etc), to estimate the residue-based local flexibility in the apo and holo states (see Materials and Methods). By evaluating the mobility difference between the holo and apo complexes, the dynamical modulation induced by sm27 on the nanosecond scale is defined.

The comparison of MD local flexibility data and experimental ^1^H→^15^N NOE data for FGF2 in the apo state and in the complex with **sm27** molecule shows that there is a high degree of correlation between the dynamical data obtained by simulations and the ones obtained via NMR. The degree of correlation is −0.7 for the apo state and −0.75 for the holo state when the calculation is carried out over the whole microsecond trajectories.

**Figure 3:**
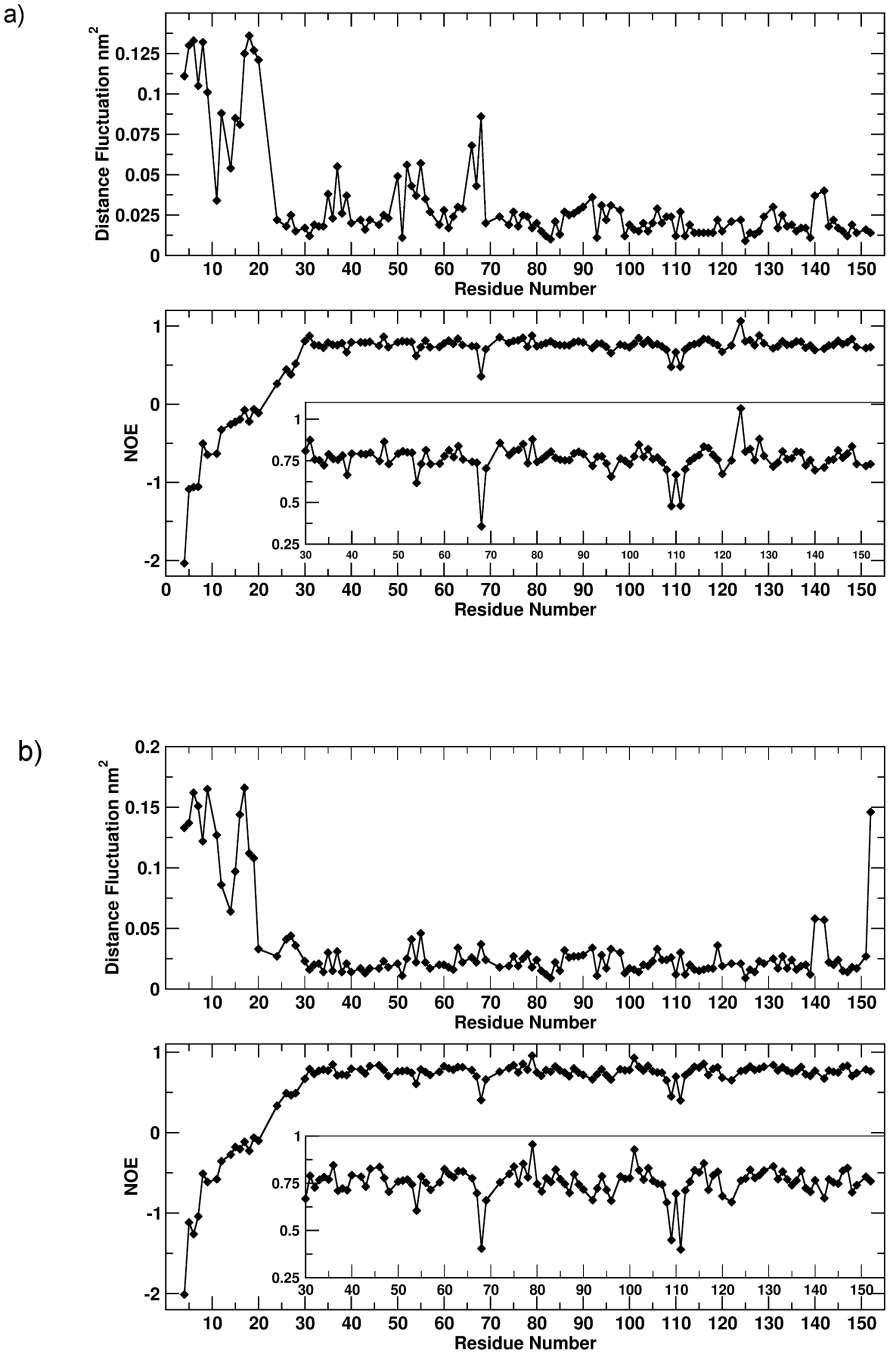
Comparison between the experimental and calculated local dynamics. a) Comparison between local dynamics for the apo system, calculated over the 100 ns trajectory interval yielding the best match (800–900 ns). b) Comparison between local dynamics for the holo system, calculated over the 100 ns trajectory interval yielding the best match (200–300 ns). The insets show a magnification of the graphs to improve clarity.

Carrying out the same analysis on all the sub-trajectories obtained by partitioning the microsecond long runs into consecutive 100 ns intervals identifies ensembles that show better correlations. For the apo state this corresponds to the interval between 800 and 900 ns (with a correlation of −0.8) (**Figure 3a**). Interestingly, in the holo state, the interval 200–300 ns shows once more a non-negligible (anti)correlation of −0.81. This result corroborates and supports the observation previously reported for *S^2^* calculations, indicating that the ensemble of structures visited in the 200–300 ns time-interval is the one that best recapitulates the structural dynamic properties of the complex (**Figure 3b**).

Taken together, the data presented in this and in the previous paragraph indicate that a specific dynamic substate obtained from the long MD runs can provide a set of conformations in dynamic exchange that overall account for the NMR-determined dynamic parameters: in particular, such ensemble may be used to visualize changes in the poses of the ligand and in the orientations of important functional groups that reverberate in the observed modulation of the dynamics of FGF2.

### Representative ensemble of FGF2/sm27 complex

Prompted by the above reported observations, we selected the ensemble of protein-ligand complex structures that could recapitulate the best match between the MD and NMR-based measurements of internal motions. This is a necessary step in view of using dynamics based information to design improved ligands. The availability of structural models combined to the analysis of the distribution of, e.g., the distances between the side-chains involved in recognition can suggest different points of intervention for the modification of the ligand, or help develop pharmacophore models in which the distributions of distances among critical functionalities are used to define upper and lower boundaries for geometric constraints.

**Figure 4:**
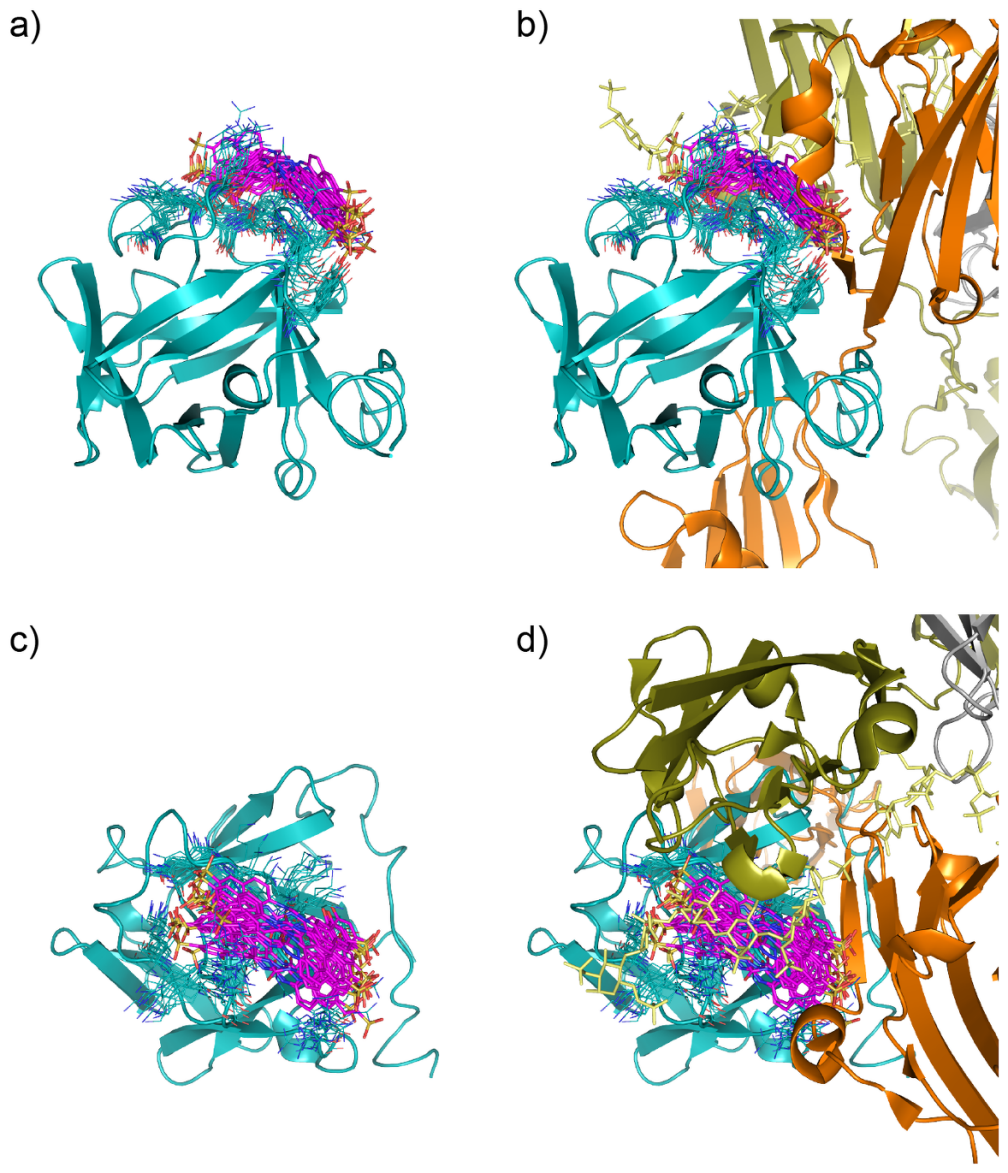
Representative structures of the ensemble of the sm27/FGF2 complex that recapitulates calculated and experimental data. From structural representations of two different orientations of the **sm27**-FGF2 complex (a and c) it is immediately evident that the ligand can occupy a diverse range of conformations/configurations and the protein dynamically adapts to them. Sm27 molecule is shown in magenta sticks, while protein side-chains involved in interaction (Y33, K35, N36, R53, K128, R129, K134, Q143, K144, A145) are shown as blue lines. In b and d the same views of sm27-FGF2 complex are offered and the position of heparin (yellow sticks) and FGFR1 (dark olive and orange cartoons), deduced from X-ray structure (PDB ID: 1FQ9), are shown.

**Figure 5:**
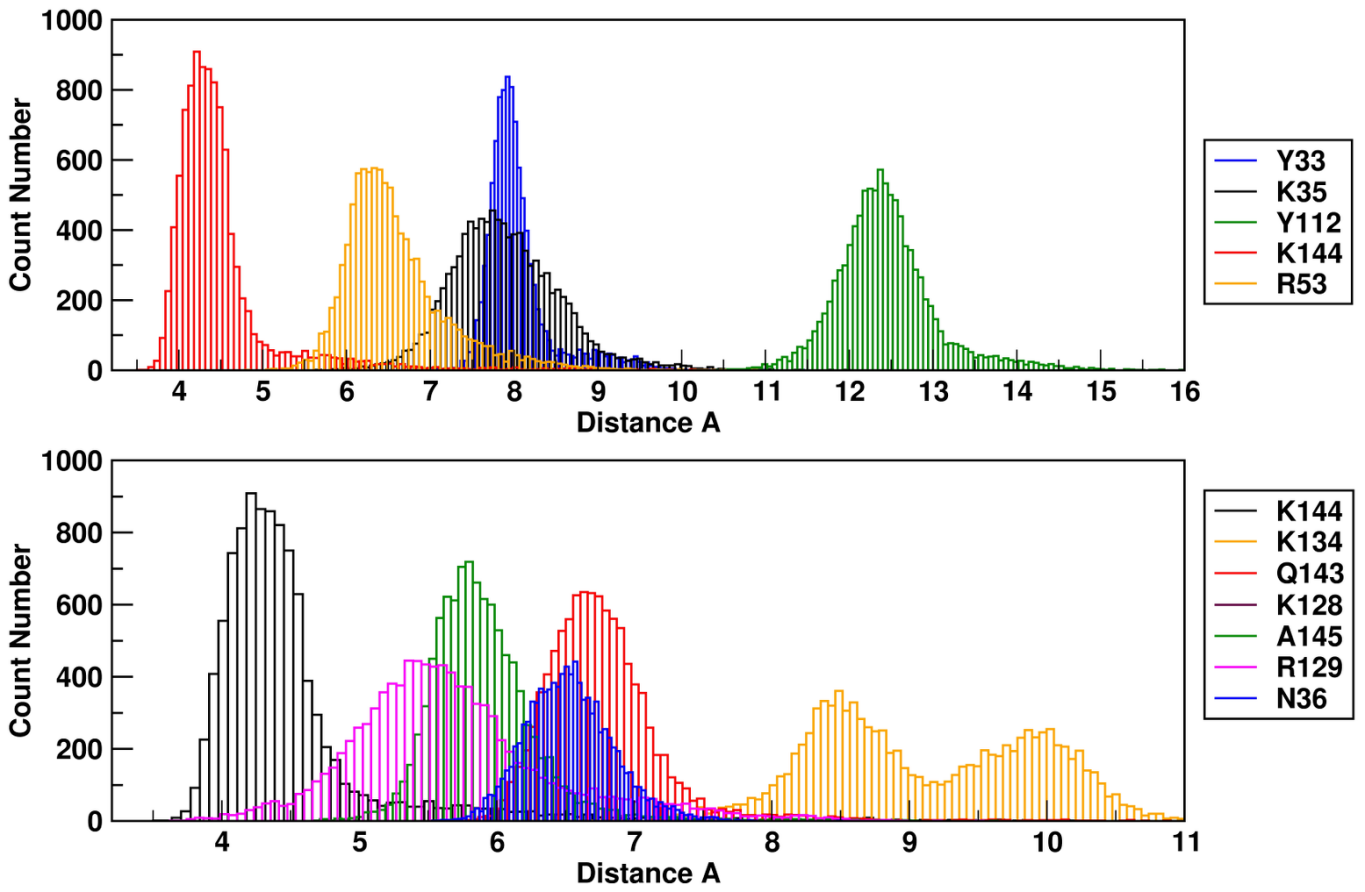
Distances between FGF2 binding site aminoacids and sm27. Histogram of the distance between the COM of the ligand calculated over the 200–300 ns interval.

We thus focused on the ensemble of representative conformations, corresponding to the simulation interval between 200 and 300 ns, which is depicted in **Figure 4**. The selected structural ensemble shows that **sm27** preferentially engages the protein surface responsible for interaction with heparin and FGFR receptor. The ligand can sample different relative positions, which may partially differ from an initial single-structure model obtained to satisfy specific restraints obtained from chemical shift perturbations [14]. Specifically, our data show that **sm27** moves on the surface of FGF2, while conserving the main contacts with the protein side chains that are needed to ensure productive and stable binding. **Figure 5** shows the distribution of the distances between the center of mass (COM) of the ligand and the side-chains of the main contacting residues, plotted as histograms. In this case the distribution is more suitable for characterizing an ensemble of conformations. Based on the inspection of the starting structure of the complex, we monitored along the MD simulation the distance between **sm27** and the residues that appeared to be close to the ligand in the starting structure (distance within 6.5 Å), namely residues Y33, K35, N36, R53, Y112, K120, R129, K134, Q143, K144, A145. Figure 5 (combined to Figure 4) indicates that the different residues can populate conformations yielding different types of distance distributions. Among them K35, N36, R129, K144 and A145 display distributions centered at shorter values, lower than 8 Å. For some of them the relevant contribution in ligand recognition suggested by MD trajectory analysis is supported by experimental data. Specifically, R129 and K144 backbone resonances were determined to be the most responsive to the presence of the molecule, based on NMR chemical shift perturbation analysis. In addition changes in the HSQC peak intensity, as a function of **sm27** additions, were observed for N36 and A145, suggesting that binding affected their dynamic behavior. The potential of the present MD approach is to highlight the involvement of additional residues in drug recognition which were not clearly picked up by NMR analysis, possibly due to the transient involvement in recognition and/or to the longer interatomic distances which make negligible the measurable effect on the resonance.

This variety of possible structural combination would not be caught by a single structure representation. In this context, the analysis of MD simulations presented here provides information on the weight and relevance of the possible interaction of residues involved in binding and **sm27**, information that may be proficiently used in pharmacophore feature selection and design.

### Protein hydration

Finally, we evaluated the distribution of water molecules in the complex. Water plays a key role both in determining molecular recognition mechanisms and in determining the overall flexibility of proteins.

**Figure 6:**
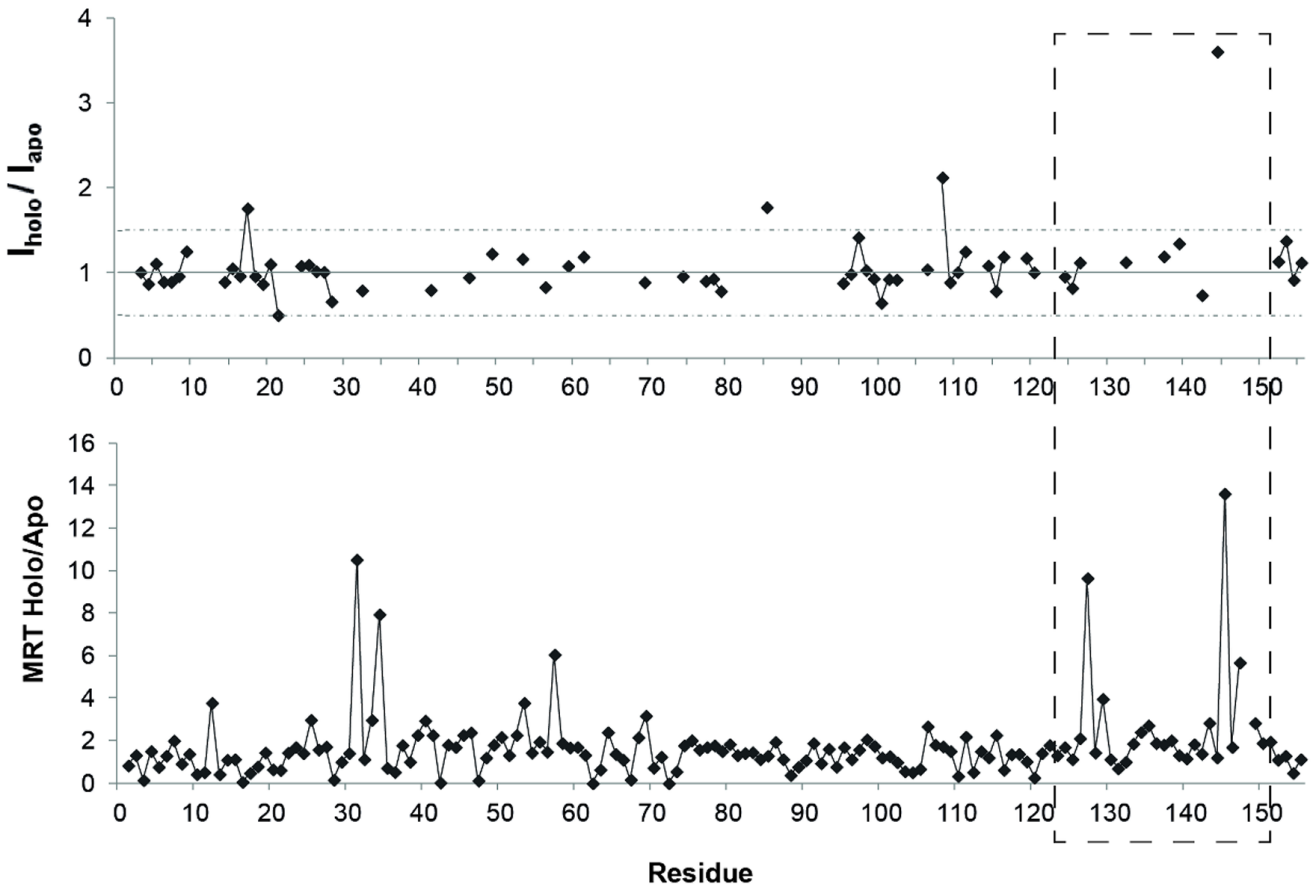
FGF2 hydration changes induced by Sm27 binding. Upper panel: ratio of the normalized intensities observed for holo and apo FGF2 is reported as a function of the residue number as derived from ePHOGSY NOE spectra. Dotted lines delimit intensity variations beyond ±50% upon **sm27** binding. Lower panel: Ratio of the mean residence time (MRT) per residue calculated for all atoms over the whole MD trajectories of apo and holo protein. Distance cut-off of 3.5 Å was used. Dashed gray line highlights the binding site regions.

**Figure 7:**
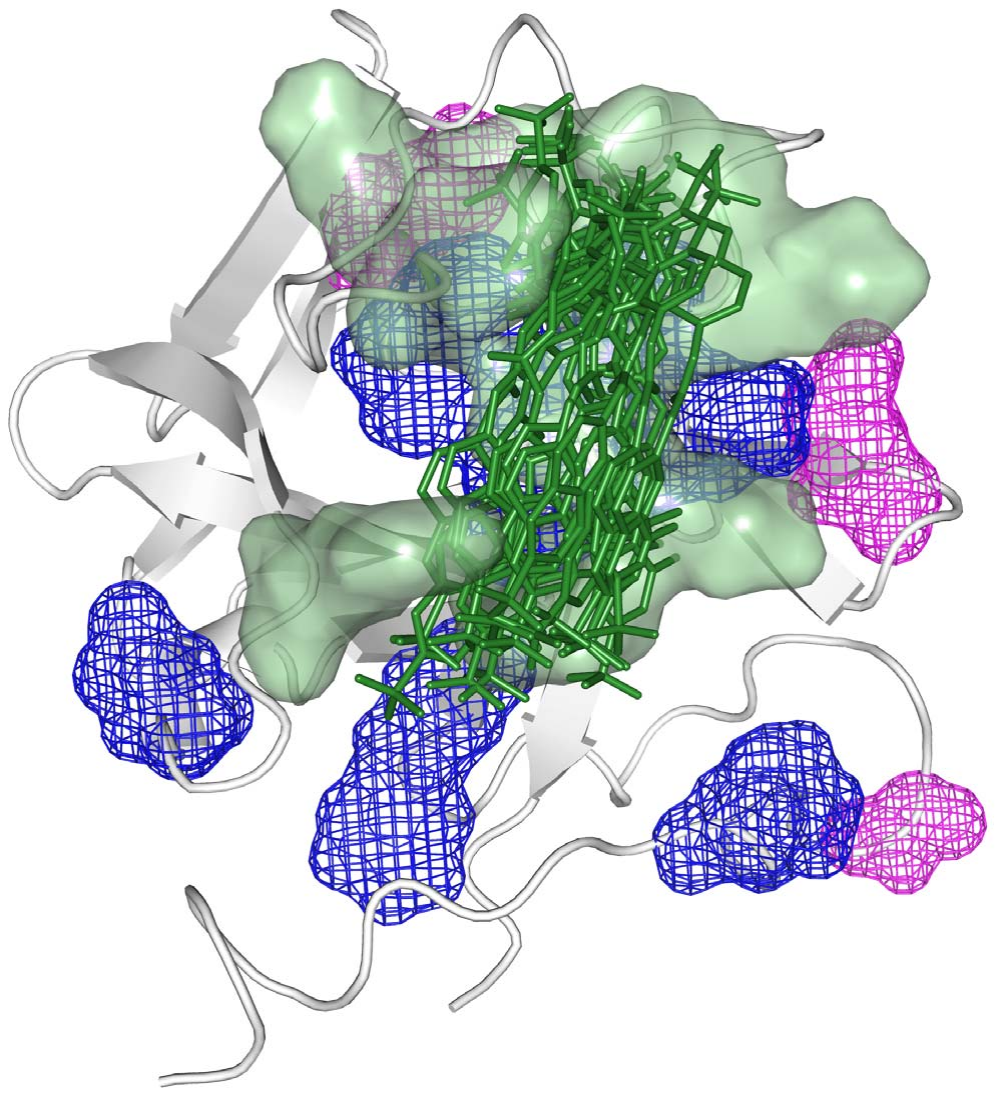
FGF2 hydration changes induced by sm27 binding. Residues affected by hydration changes upon **sm27** binding, as deduced from MRT analysis (blue) and ePHOGSY NMR data (magenta) are shown as mesh. Residues Y33, K35, N36, R53, K128, R129, K134, Q143, K144, A145, involved on the average in the contact with the ligand (green sticks), are shown as green surface.

NMR spectroscopy is a well-suited technique to assess the presence of bound waters through ePHOGSY experiments (enhanced Protein Hydration Observed through Gradient SpectroscopY). In this kind of spectra peak intensities depend on distance between water and protein protons, on protein dynamics, and on the residence time of water on the protein [32]. Previous NMR studies suggested a change in the hydration state of FGF2 upon **sm27** binding, mainly localized at the level of the N-terminal segment and of the **sm27** binding site (K144) [14]. In Figure 6 the comparison of the binding effects on FGF2 hydration, as deduced from NMR and MD, is reported. Interestingly, the regions that are most prone to show hydration changes upon apo to holo transition, also correspond to the regions that responsive to the presence of the ligand, in terms of local and long-range changes in dynamics. MRT analysis is able to pick out the change of hydration at the level of residues close to the **sm27** binding site (residues 127, 129, 145 and 147) and this change is supported by NMR data. Additional residues, showing a significant variation upon binding as deduced from MD analysis (12, 31, 34, 57) do not exactly match the residues highlighted by NMR analysis (17, 85, 108), however they lay on a common FGF2 face (Figure 7).

### The importance of dynamics in the structural description of ligand protein complexes, and its implications for drug-design

Our understanding of receptor-ligand binding mechanisms in biology has significantly progressed from the initial lock-and-key model. It is now recognized that molecular recognition between interacting molecules is a dynamic process and proteins are not static entities: they fluctuate among a number of states on their accessible energy landscape and exploit this dynamics to select specific conformations related to functional tasks. In this context, the ligand can choose the binding partner among available states, eventually shifting the population distribution [33]–[37]. The dynamic nature of molecular recognition can thus impact on the structural and conformational properties of both partners. In drug discovery and design the characterization of the dynamic interplay between a protein and a ligand can clearly provide new opportunities, particularly in the field of the development of inhibitors of protein-protein interactions. In this case, the target site on the protein is often a large, flat and flexible surface, onto which the ligand can adapt in a multiplicity of possible poses and conformations. These aspects are particularly important in the biophysical characterization of FGF interactions, in the understanding of its interactions with the receptor and in the elucidation of its cellular activities [38].

The characterization of the ensemble of representative poses can aptly indicate the most probable interactions, as well as their relative importance, between the ligand's functional groups and specific protein residues, which might not be identified using a single-structure representation of the complex. Moreover, the analysis of the modifications in protein structures in response to ligand binding can assist in defining chemical modifications on the ligand that favor optimal binding. Finally, the information on the conformational distribution of relevant side chains on the receptor could be used to develop pharmacophore models for virtual screening, keeping into account the motional and flexibility properties of both the ligand and the receptor. The distributions of the distances between the ligand and binding site residues can indeed be used to define upper and lower boundaries for geometric constraints.

In this work, we have used a combined approach to select the ensemble of ligand-protein complex conformations from MD simulations that best match the structural and dynamic profiles obtained from NMR analysis. The net result is the possibility to filter a conformational ensemble for the FGF2-**sm27** complex out of a microsecond long simulation, that quantitatively fits the structural, flexibility, and hydration parameters obtained by NMR experiments on both the apo and holo states of the protein.

The representative ensemble of conformations, corresponding to the simulation interval between 200 and 300 ns, is depicted in Figure 4 and Figure 7. The selected structural ensemble shows that **sm27** preferentially engages the protein surface responsible for interaction with heparin and FGFR receptor and can sample different relative positions, which may partially differ from an initial single-structure model obtained to satisfy specific restraints. In this context, the analysis of the dynamic cross-talk between the protein and the ligand can be exploited to characterize the formation/disappearance of pockets in the interaction region. This information can then be productively exploited to modify the initial lead **sm27** with novel appendages and functional groups that are selected to optimize the fit within the dynamic binding site (see surface representation in Figure 7). We are currently pursuing this strategy and the newly designed derivatives will be evaluated for their antiangiogenic properties in vivo and in vitro.

In conclusion, this study has been primarily aimed at investigating the dynamics of a small molecule ligand on a flat, exposed protein surface, representing a typical target in the development of protein-protein interaction inhibitors. We have approached this topic using microsecond MD simulations and NMR-based characterizations of the dynamics of the apo and holo states of the system. Using a direct comparison of parameters from the two techniques, we have filtered out the set of conformational states that are used to generate a multi-structure representation of the complex. This may be an important step in recapitulating and describing molecular recognition events, in particular for systems where the mutual dynamic influence between the interacting partners is expected to play an important role. Our approach can be a viable method for obtaining insights into the protein and ligand dynamics in drug design and development, in particular in the context of protein-protein interactions. The results presented here also provide the potential to guide the expansion of the chemical diversity space of FGF2 inhibitors with a rational approach.

## Supporting Information

File S1 **Figures S1–S3.** Figure S1. The chemical structure of sm27. Figure S2. ^15^N-spin relaxation parameters of FGF2. Figure S3. Model-free parameters.
(PDF)
