# Supplementary material for: Investigating the Dynamic Aspects of Drug-Protein Recognition through a Combination of MD and NMR Analyses: Implications for the Development of Protein-Protein Interaction Inhibitors
Source: PLoS One. 2014 May 27;9(5):e97153. doi: 10.1371/journal.pone.0097153 (PMC4035249; doi:10.1371/journal.pone.0097153)
Supplement: File S1 — Figures S1–S3. Figure S1. The chemical structure of sm27. Figure S2. 15N-spin relaxation parameters of FGF2. Figure S3. Model-free parameters. (PDF) [file pone.0097153.s001.pdf]

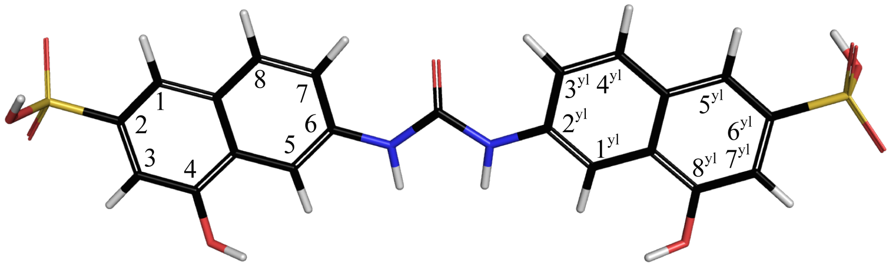

Figure S1. Chemical structure of **sm27.**

Supplementary Materials

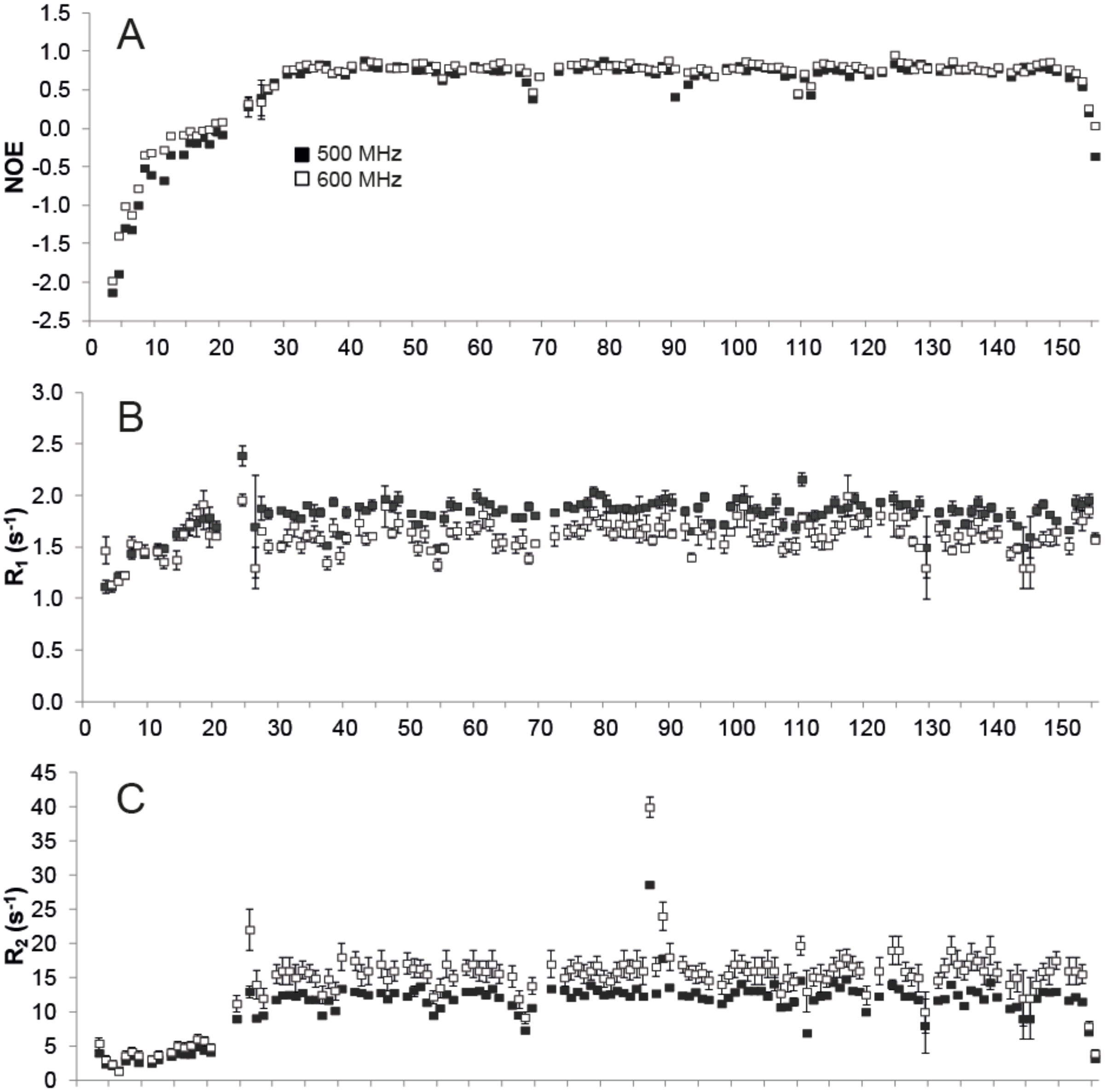

Figure S2 ^{15}N-spin relaxation parameters of FGF2 measured at 500 MHz and 600 MHz: (A) Steady-state heteronuclear NOE, (B) R2, (C) R1, and (D) R2/R1 values.

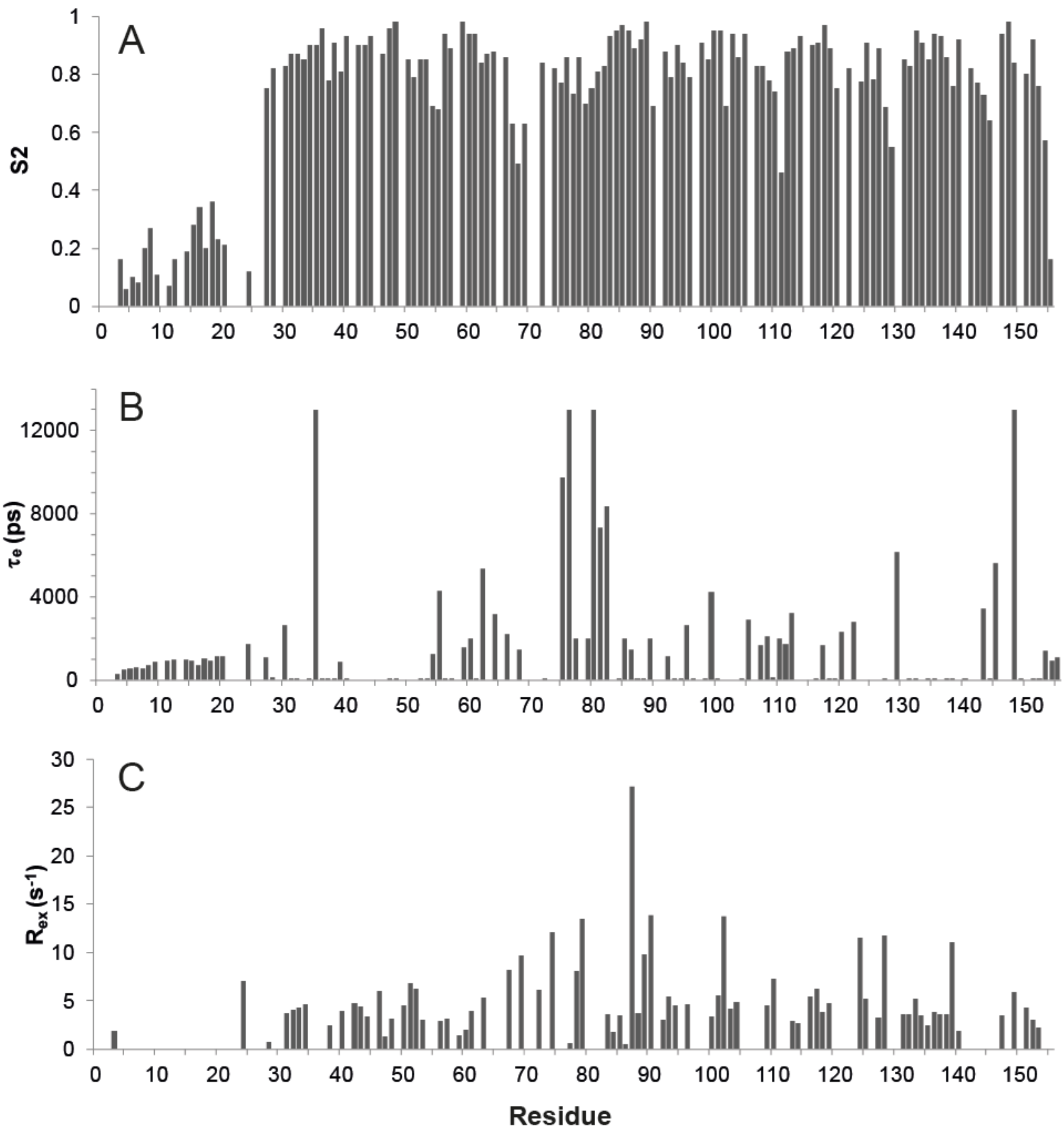

Figure S3 Model-free parameters. (A) S2, (B) te, and (C) Rex of FGF2 backbone amides, derived from a model-free analysis of the relaxation data at 500 and 600 MHz.
